# An Observational Study Comparing Hybrid Transvaginal Notes and Four-Port Laparoscopic Cholecystectomy

**DOI:** 10.7759/cureus.33589

**Published:** 2023-01-10

**Authors:** Asif M Ansari, Gourav Kaushal, Kanwarjit S Dhillon

**Affiliations:** 1 General Surgery, Vishwakarma Hospital and Heart Care Center, Sambhal, IND; 2 Surgical Gastroenterology, All India Institute of Medical Sciences, Bathinda, IND; 3 General and Laparoscopic Surgery, Max Hospital, Mohali, IND

**Keywords:** cholecystectomy, notes, hybrid notes cholecystectomy, laparoscopic cholecystectomy, cholelithiasis

## Abstract

Background: Recently, a great interest has arisen in hybrid natural orifice transluminal endoscopic surgery-cholecystectomy (NOTES-C). It has the potential to cause less postoperative pain and may offer better cosmesis over conventional laparoscopic cholecystectomy (CLC).

Patients and methods: A total of 112 females who underwent conventional cholecystectomy were compared with 108 patients of hybrid transvaginal NOTES-cholecystectomy (TV NOTES-C). We compared intraoperative factors, postoperative pain, the analgesic requirement at different intervals, duration of hospital stay, and time to return to normal activities. In addition, cosmesis and patient satisfaction were assessed at four weeks.

Results: Postoperative pain and analgesic requirement were less in the hybrid TV NOTES-C group (p<0.001 at 95% CI). Hybrid TV NOTES-C patients were discharged more frequently within 12 hours (27.5% versus 1.8%; p<0.001) and returned faster (2.22 versus 4.62 days; p<0.001) to normal activities. Cosmetic results and short-term quality of life as assessed by the patient and observer scar assessment scale (POSAS) and short form-36 (SF-36) scores, respectively, were better in the hybrid TV NOTES-C group (p<0.001 at 95% CI).

Conclusions: Hybrid TV NOTES-C is associated with reduced postoperative analgesic requirements, faster return to normal activities, better cosmesis, and patient satisfaction compared to conventional four-port cholecystectomy.

## Introduction

Open cholecystectomy was a standard procedure for removing the gallbladder for decades. However, it is well known that large abdominal incisions are associated with more pain, wound infections, incisional hernias, and ugly scars. Therefore, since the last decade of the 20th century, four-port laparoscopic cholecystectomy, also known as conventional laparoscopic cholecystectomy (CLC), has gradually replaced open cholecystectomy as the preferred approach for gallstone disease [1]. Compared to open cholecystectomy, CLC is associated with decreased postoperative pain and need for postoperative analgesia, shortened hospital stay, early return to work, improved cosmetic outcomes, and better patient satisfaction [2].

Natural orifice transluminal endoscopic surgery (NOTES) has been attracting the attention of many surgeons lately. Compared with the conventional laparoscopic procedure, the new NOTES technique has the potential to minimize abdominal wall incisions, minimize wound-related complications, reduce postoperative pain, and lead to better cosmetic outcomes. In this technique, the target organ in the abdominal cavity is accessed through natural orifices, such as the mouth, vagina, bladder, or anus [3,4].

For many decades, gynecological procedures have been performed through natural orifice, i.e., transvaginal [5-7]. Even in the late 20th century, the transvaginal route was used to deliver the gallbladder with large stones and other intraabdominal organs [8,9]. In 2004, Kalloo et al. proposed performing NOTES for gastroenterological surgeries and used transgastric liver biopsies in the porcine model [10]. In pure NOTES, there is no instrument entry done from the abdomen. However, even with modern, flexible endoscopes, pure NOTES techniques are challenging, take significantly longer time, and are associated with more complication rates. Thus, several authors suggested hybrid transvaginal NOTES (transvaginal+transabdominal) route to overcome the limitations of pure NOTES [11-15].

There are a few randomized studies for NOTES cholecystectomy. The NOVEL trial, prospective, randomized comparison between natural orifice and laparoscopic cholecystectomy demonstrated the safety profile of the transvaginal approach along with superior cosmesis and decreased pain [16]. However, there is a dearth of literature evidence for or against this new surgical approach.

In this prospective observational study, we have compared conventional four ports laparoscopic and transvaginal hybrid NOTES cholecystectomy. The study aimed to compare the two techniques for postoperative pain scores, analgesic requirements, and cosmetic outcomes. The two groups were also compared for the length of the hospital stay, port site complications, time taken to return to normal activities, and patient satisfaction.

## Materials and methods

This prospective comparative observational study was conducted in the Department of General and Gastrointestinal (GI) Surgery, Max Superspeciality Hospital, Mohali, Punjab, India, from October 2017 to July 2018 (IRB approval number TS/MSSH/MOHALI/HEPL/IEC/GENSUR/17-02). Two hundred twenty patients who underwent these two procedures for symptomatic uncomplicated cholelithiasis during the study period were included. Group A comprised 112 patients who underwent conventional laparoscopic cholecystectomy (CLC), and group B comprised 108 patients who underwent hybrid transvaginal NOTES-cholecystectomy (TV NOTES-C).

Inclusion and exclusion criteria

Consenting females aged 18 years and above with symptomatic uncomplicated cholelithiasis were included in the study. Patients with active pelvic infection (PID), patients having severe endometriosis, severe pelvic or upper abdominal adhesions, morbid obesity (BMI>40 kg/m^2^), pregnant females, and sexually non-active/virgin females were excluded from the study.

Data collection methods

The patient selected the surgical technique after understanding both techniques' possible advantages and disadvantages and the cost differences. Patients were admitted on the day of surgery, and demographic data and patient characteristics were recorded in the standard proforma.

Surgical techniques

**Figure 1:**
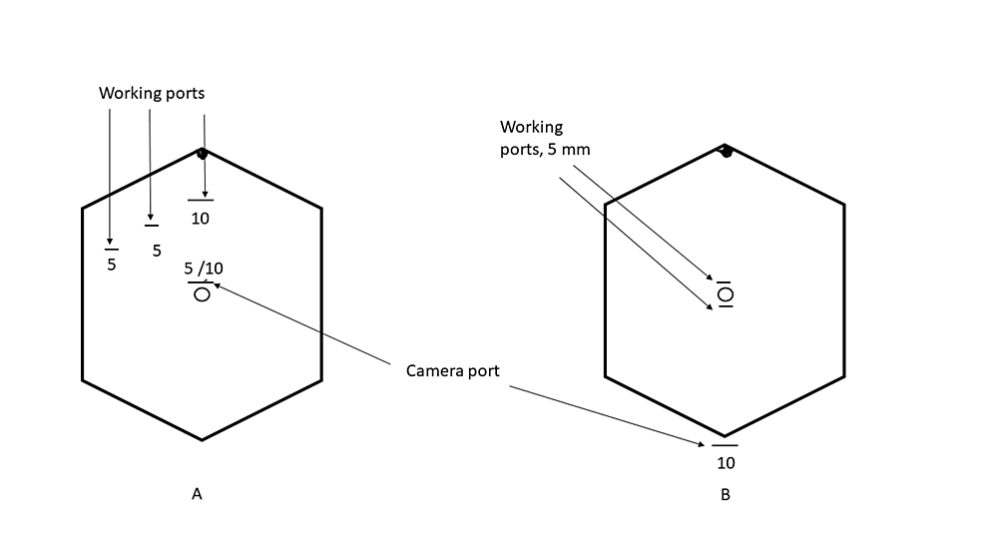
Port positions in conventional (A) and hybrid transvaginal NOTES cholecystectomy (B). NOTES: natural orifice transluminal endoscopic surgery

CLC was performed using a standard four-port technique. Hybrid TV NOTES-C was performed by the method earlier published by Dhillon et al. in 2017 [17]. Ports insertion sites in both techniques are illustrated in Figures 1A, 1B.

Intraoperative findings like operative time and complications (including other visceral injuries) were noted. Postoperatively pain score using a visual analog scale (VAS) was noted at 6, 12, and 24 hours, on postoperative day (POD) 4 and POD 7. The need for analgesics, type, dose, and frequency was recorded at similar intervals. After four weeks, pain score, port site complications, cosmetic assessment (patient and observer scar assessment scale {POSAS}), and patient satisfaction (short form-36 {SF-36}) were assessed. The data was stored in a Microsoft Excel sheet and analyzed using IBM SPSS version 21 (Armonk, NY: IBM Corp.). The chi-square test was the test of significance for qualitative data. A p-value <0.05 was considered statistically significant at a 95% confidence interval.

## Results

The study consisted of 112 patients (group A) of CLC and 108 (group B) of hybrid TV NOTES-C. The mean age (SD) of groups A and B were 50.85 (14.06) and 43.44 (12.91) years, respectively. The most common presenting symptom at inclusion was pain, followed by dyspepsia and nausea with or without vomiting. Most of the patients had overlapping symptoms.

Intraoperative observations

There was no biliary injury or other visceral injuries during the study period. There were no conversions to open surgery. The mean duration of surgery in the hybrid TV NOTES-C group was 36.35±9.1 minutes and in cases of conventional laparoscopic cholecystectomy was 48.15±9.9 minutes. The duration was significantly less in cases of the hybrid TV NOTES-C group (p<0.01).

Postoperative observations

Pain: Postoperative mean pain scores (VAS) were 5.84±1.35, 4.88±1.18, and 4.07±1.12 after 6, 12, and 24 hours, respectively, for the CLC group. Scores for the hybrid TV NOTES-C group were 4.01±1.19, 2.46±0.83, and 1.68±0.71, respectively, at similar intervals. It was significantly better (p<0.001) for the hybrid TV NOTES-C group at every interval (Table 1).

**Table 1:** Postoperative pain scores. CLC: conventional laparoscopic cholecystectomy; TV NOTES-C: transvaginal natural orifice transluminal endoscopic surgery-cholecystectomy; VAS: visual analog scale; POD: postoperative day

VAS	CLC	Hybrid TV NOTES-C	p-Value
6 hours	5.84±1.35	4.01±1.19	<0.001
12 hours	4.88±1.18	2.46±0.83	<0.001
24 hours	4.07±1.12	1.68±0.71	<0.001
POD 4	0.99±0.36	019±0.39	<0.001
POD 7	0.27±0.60	0.04±0.23	<0.001

The injectable analgesic (paracetamol, diclofenac, and tramadol) requirement after 6, 12, and 24 hours of surgery were compared and was found to be significantly less in group B at 6 hours (Table 2). At 12 and 24 hours, analgesic requirements were significantly less in group B than in group A. Postoperatively pain scores on POD 4 and POD 7 were 0.99 versus 0.19 and 0.27 versus 0.04 for groups A and B, respectively (Table 3). 

**Table 2:** Analgesics requirement postoperatively at 6 hours. CLC: conventional laparoscopic cholecystectomy; NOTES-C: natural orifice transluminal endoscopic surgery-cholecystectomy; PCM: paracetamol; O: oral; I: injection; A: aceclofenac

Variables	CLC	Hybrid TV NOTES-C	p-Value
PCM-O 650 mg	100%	100%	1
PCM-I 900 mg	45.5%	11%	<0.001
A+PCM (100 mg + 325 mg)-O	100%	100%	1

**Table 3:** Scar assessment and patient satisfaction after four weeks of surgery. CLC: conventional laparoscopic cholecystectomy; NOTES-C: natural orifice transluminal endoscopic surgery-cholecystectomy; POSAS-P/O: patient and observer scar assessment score-patient/observer

Variables	CLC	Hybrid TV NOTES-C	p-Value
POSAS-P	23.56±2.52	12.03±1.16	<0.001
POSAS-O	19.16±1.05	7.94±0.82	<0.001
SF-36	14.36±1.59	3.90±0.73	<0.001

Discharge from the hospital and follow-up observations

On the same day, 1.8% of patients in Group A and 27.5% of patients in Group B were discharged (p<0.001). Rest of the patients were discharged on the next day of surgery. During follow-up visits, patients were asked about the number of days taken to return to the regular pre-surgery schedule. Group A patients, on average, took 4.62 days compared to 2.22 days for group B patients. This difference was significant (p<0.001).

Follow-up After Four Weeks

**Figure 2:**
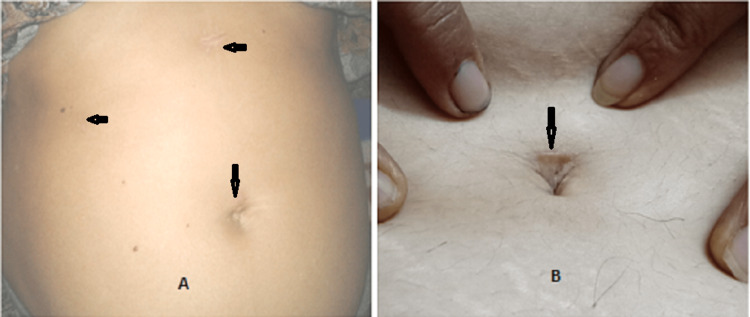
Scar appearance after four weeks following CLC. Arrows pointing towards the port site scars, it's notable that lumbar port scar is hardly visible (A) and hybrid TV NOTES-C, another scar at the lower margin of the umbilicus is not visible (B). CLC: conventional laparoscopic cholecystectomy; TV NOTES-C: transvaginal natural orifice transluminal endoscopic surgery-cholecystectomy

All patients were given a questionnaire for scar assessment and overall satisfaction (Table 3). For scar assessment, the "patient and observer scar assessment scale-patient/observer (POSAS-P/O)" was used, and for overall satisfaction, a "Short form-36 (SF-36)" questionnaire was used. POSAS-P, POSAS-O, and SF-36 scores were 23.56, 19.16, and 14.36, respectively, for group A patients. For group B patients, these scores were 12.03, 7.94, and 3.90, respectively. All scores were significantly better for group B (p<0.001). After four weeks, the cosmetic appearance of CLC and hybrid TV NOTES-C scars are shown in Figures 2A, 2B. None of the patients had incision or port-site infection and port-site incisional hernia after a follow-up period of four weeks.

## Discussion

We observed that the mean age of the patients was 50.26 years and 43.72 years for CLC and hybrid TV NOTES-C groups, respectively. It indicates that most young female patients preferred hybrid TV NOTES-C surgery; the difference was significant. Dhillon et al. in 2017 [17] compared the present technique of hybrid NOTES cholecystectomy with existing techniques published by Ramos et al. [18], Roberts et al. [19], Zornig et al. [11], and Forgione et al. [20] and reported its safety and feasibility.

Our study noted that operating time was significantly less for the hybrid TV NOTES-C group than for the CLC group (36.35 versus 48.15 minutes; p<0.01). Our findings contradict existing literature which suggests that hybrid TV NOTES-C is a more time-consuming surgery [21,22]. We also expected hybrid TV NOTES-C to take more time, a more complex and uncommon technique. Unpredicted results might be because all hybrid TV NOTES-C were performed by the single and most experienced surgeon, who has >1000 NOTES experience, and CLCs were performed by multiple surgeons and surgical residents.

Our results are comparable with a meta-analysis of nine studies by Xu et al. regarding pain scores and analgesic requirements in the postoperative period [23]. It showed that pain scores and analgesic requirements were significantly lower in hybrid TV NOTES-C compared to CLC. Our comparative study observed that 27.5% of patients in the hybrid TV NOTES-C group and 1.8% in the CLC group were discharged on the same day of surgery (<12 hours). This difference was also significant. Our results are comparable to the case series presented by Noguera et al., in which nine and two out of 15 patients were discharged in <24 and <12 hours, respectively [24].

In our study, hybrid TV NOTES-C group patients took 2.22 days to return to normal activities, and the CLC group took an average of 4.62 days (p<0.001). Similar results were shown in a systematic review and meta-analysis of 14 studies describing 1145 patients [25].

In this study, we have compared cosmetic outcomes using the patient and observer scar assessment scale (POSAS-P/O) by the patient herself and the observer in the clinic. In addition, we have analyzed patient satisfaction by using the SF-36 questionnaire. After four weeks of surgery, scar assessment and patient satisfaction were significantly better in cases of hybrid TV NOTES-C group patients. Our results concur with the previously published studies, suggesting better cosmesis [17,26] and patient satisfaction following hybrid transvaginal NOTES cholecystectomy [19,27]. 

In our series, we did not get any incisional/port-site hernia after a follow-up of four weeks, but it is well known that the incidence of an incisional hernia at the port-site is approximately 1.7% after a longer follow-up [28]. Therefore, favorable results in our series are likely due to a short follow-up.

There are two major pitfalls in this study. First, the present study is not randomized or adequately powered; hence, results must be interpreted cautiously. Second, it includes different surgeons with differing experiences doing surgeries, and one surgeon exclusively performed NOTES procedures; it has likely led to lesser operative time for the NOTES technique. Further, adequately powered pragmatic and randomized trials are required to substantiate the safety and feasibility of hybrid TV NOTES-C.

## Conclusions

Hybrid TV NOTES-C is a feasible, safe, and acceptable technique for gallbladder removal. In addition, hybrid TV NOTES-C causes less postoperative pain and has reduced postoperative analgesic requirements allowing early resumption of regular activity. It also offers better cosmetic outcomes and patient satisfaction compared to CLC. Larger, pragmatic trials comparing these modalities may help in wider acceptance of hybrid TV NOTES technique.
